# TE optimization for J‐difference editing of 2‐hydroxyglutarate at 3T

**DOI:** 10.1002/mrm.30561

**Published:** 2025-05-20

**Authors:** Kimberly L. Chan, Rutul Hapani, Elizabeth A. Maher, Toral R. Patel, Anke Henning

**Affiliations:** ^1^ Advanced Imaging Research Center, University of Texas Southwestern Medical Center Dallas Texas USA; ^2^ Department of Psychiatry University of Texas Southwestern Medical Center Dallas Texas USA; ^3^ Department of Biomedical Engineering University of Texas Dallas Dallas Texas USA; ^4^ Department of Internal Medicine University of Texas Southwestern Medical Center Dallas Texas USA; ^5^ Department of Neurology UT Southwestern Medical Center Dallas Texas USA; ^6^ Department of Neurological Surgery University of Texas Southwestern Medical Center Dallas Texas USA; ^7^ Department of Radiology University of Texas Southwestern Medical Center Dallas Texas USA

**Keywords:** 2HG, glioma, J‐difference editing, MR spectroscopy, tumor

## Abstract

**Purpose:**

To investigate the TE dependence of the edited 2‐hydroxyglutarate (2HG) signal, its separation from co‐edited glutamate plus glutamine (Glx), and fit accuracy in the presence of nuisance signals using a MEGA‐PRESS sequence.

**Methods:**

Simulations were performed at TEs 70–160 ms to assess the signal intensity and 2HG‐Glx overlap as a function of TE. The effect of the 2HG‐Glx spectral overlap on the fit accuracy of 2HG was evaluated on simulated 2HG‐edited spectra with in vivo parameter variations. Data were acquired at TEs of 70 and 90 ms in 13 glioma patients to estimate the TE‐dependence of the 2HG and Glx signal intensity and at a TE of 120 ms in eight glioma patients to estimate the in vivo 2HG, Glx, and water T2 relaxation times.

**Results:**

A TE of 90 ms was found to produce a maximal 2HG integral, which was 23% larger than that at a TE of 70 ms in vivo without a significant increase in 2HG‐Glx overlap. Lipid and residual water were 26% and 16% lower, respectively, at a TE of 90 ms versus 70 ms. Fit‐quality numbers were 49% lower at a TE of 90 ms versus 70 ms, indicating enhanced fits at a TE of 90 ms. The in vivo T2 relaxation times of 2HG, Glx, and water were 264, 177, and 110 ms, respectively.

**Conclusion:**

A TE of 90 ms was best with a maximal 2HG signal, minimal 2HG‐Glx overlap, and minimal residual water and lipid contamination.

## 1 INTRODUCTION

Mutations in the genes isocitrate dehydrogenase (IDH) 1 and 2 are present in 80% of low‐grade gliomas^1^, ^2^ and result in abnormal brain metabolism where α‐ketoglutarate, an intermediate product of the tricarboxylic acid cycle, is converted to 2‐hydroxyglutarate (2HG).^3^ This excess 2HG production can reach millimolar concentrations in IDH‐mutant tumors and can be detected in vivo with ^1^H‐MRS.^4^, ^5^ Clinically, these tumors are associated with longer survival and improved response to chemotherapy.^6^ Thus, the noninvasive detection of 2HG with ^1^H‐MRS has important implications for determining IDH status for prognostic purposes and monitoring response to treatment.^7^

**FIGURE 1:**
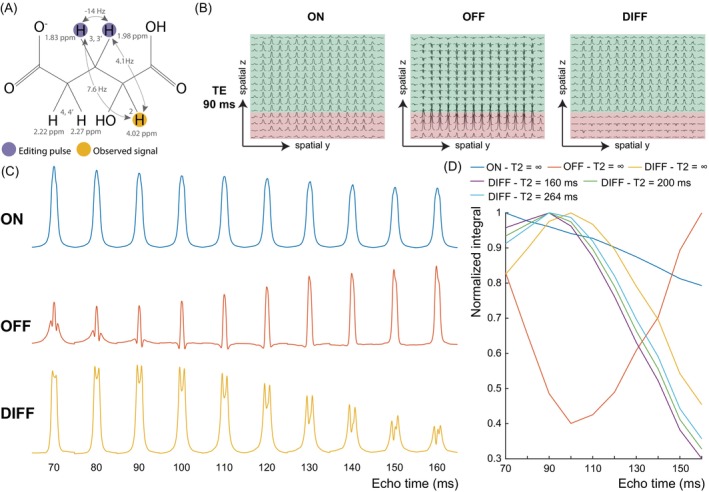
(A) Chemical structure of 2‐hydroxyglutarate (2HG) which has five non‐exchangeable protons detectable by ^1^H‐MRS. Scalar (J‐) couplings are indicated by the arrows. Editing pulses are applied at 1.9 ppm to target the moieties at 1.83 and 1.98 ppm and the observed signal originates from the proton at 4.02 ppm. (B) Spatial simulations of the 2HG multiplet at 4.02 ppm at a 90 ms for the MEGA‐PRESS sequence with 7‐Hz line‐broadening and editing pulses applied on‐resonance (ON) at 1.9 ppm (left) and off‐resonance (OFF) at 7.5 ppm (middle). The difference (DIFF) spectrum (ON – OFF) is shown on the right. Regions where coupling evolves as desired and produces a negative 2HG OFF signal are highlighted in green. Regions where couplings are refocused during the OFF acquisition are in red. (C) Edit‐ON (top), Edit‐OFF (middle), and DIFF (bottom) of the 2HG multiplet simulated with 7‐Hz line‐broadening summed over the entire voxel as a function of TE. (D) Integrals of the 2HG multiplets in (c) normalized to the maximum integral over a TE range of 70 ms to 160 ms in each acquisition type. The DIFF integrals as a function of TE are also shown with a T2 relaxation time of 160 ms and 200 ms, the upper and lower bounds of previously reported in vivo T2 relaxation times for Glx, and a T2 relaxation time of 264 ms (as estimated here from the in vivo acquisitions). The DIFF integral reaches a maximum signal intensity at a TE of 90–110 ms with no T2 relaxation, and at a TE of 90 ms with a T2 relaxation time of 264 ms, 160 ms, and 200 ms.

Due to the overlap between 2HG and other metabolites (e.g. N‐acetylaspartate [NAA]), 2HG is commonly detected using J‐difference editing to remove overlying signals and selectively detect 2HG.^8^, ^9^, ^10^ 2HG can be treated as an AMX spin system with signals at 4.02, 1.83, 1.98, 2.22, and 2.27 ppm (Figure 1A). In 2HG‐editing, the editing pulse is applied at 1.9 ppm to target the 1.83 and 1.98 ppm resonances, and an edited signal is observed at 4.02 ppm. As shown previously, the optimal TE for J‐difference editing is important for optimal detection and depends on the targeted metabolite^11^, ^12^; no such investigation has been performed for 2HG, however. Thus, 2HG‐edited studies have been acquired with various TEs including 68 ms^8^, ^9^, ^13^ and 75 ms.^10^ In theory, ignoring T2 relaxation effects, triplet‐like signals should be optimally detected at a TE of ˜1/2 J (where J is the coupling constant), and doublet‐like signals should be edited at 1/J.^11^ Assuming a typical three‐bond proton‐proton J = 7‐Hz, triplet‐like signals should be edited at ˜70 ms while doublet‐like signals should be edited at ˜140 ms. Approximating 2HG as a simple AX_2_ spin system as done previously,^10^ suggests a triplet‐like signal with optimal detection at a shorter TE of ˜70 ms. Strong coupling between H_3_ and H_3'_ brings uncertainty to this estimate, however.

Signal weightings from T2 relaxation, which bias the signal curves towards shorter TEs, also need to be considered when determining the optimal TE. The in vivo T2 relaxation constant is unknown for 2HG; however, 2HG is structurally similar to glutamate (Glu), a metabolic intermediate converted to α‐ketoglutarate during the TCA cycle that 2HG is produced from. Considering this structural similarity, 2HG likely has a similarly long T2 relaxation constant, which has been reported to be 160–200 ms at 3T for glutamate in healthy participants.^14^, ^15^, ^16^, ^17^ Thus, in vivo T2 relaxation may not affect the TE‐modulation of the 2HG signal as much as it does for short‐TE metabolites like glutathione.^11^

Another important consideration is the spectral overlap between the peak‐of‐interest and signals from co‐edited metabolites and nuisance signals such as lipids, which are elevated in gliomas,^18^ and residual water. Because of the structural similarity between Glu, glutamine (Gln), and 2HG, the edited 4.02 ppm 2HG resonance is near the co‐edited 3.75 ppm Glx (Glu + Gln) signal, resulting in partial overlap and complicated spectral fitting. As IDH‐mutant gliomas can have low tumor cellularity^5^ and, hence, low signal intensity, optimal 2HG detection by maximizing signal intensity and minimizing spectral overlap and contamination from nuisance signals takes precedence. Thus, the goal of this study was to investigate the TE‐dependence of the 2HG‐edited signal and its separation from lipid, residual water, and co‐edited Glx at 3T using simulations and in vivo data acquired with a MEGA‐PRESS sequence at multiple TEs. The T2 relaxation times of 2HG, Glx, and water in gliomas at 3T were also investigated.

## 2 METHODS

### 2.1 Simulations

The MEGA‐PRESS pulse sequence was simulated using 15‐ms sinc‐Gaussian editing pulses (bandwidth = 82 Hz) and computer‐optimized narrowband slice‐selective refocusing pulses^19^ with a bandwidth = 1264 Hz and a duration = 6.91 ms.^20^ Simulations were performed using FID‐A^21^ for a voxel size of 3 × 3 cm^2^ across a 19 × 9 matrix spanning 3.6 × 3.6 cm^2^ in the two refocusing pulse dimensions at 3T. Simulations were performed at TEs from 70 to 160 ms in 10‐ms increments and apodized with a 7‐Hz or 13‐Hz exponential filter to simulate excellent and adequate linewidths, respectively.^22^ The duration of the first slice‐selective spin echo (TE1) was kept as short as possible at 13.4‐ms to minimize signal evolution and unwanted coherences,^23^ and the timing of the second spin echo was adjusted to make up the remainder of the TE. The 2HG and Glx spectra were integrated from 3.45–4.25 ppm and summed to represent the signal for each metabolite across the simulated voxel. The simulations were performed without considering T1 relaxation, as all data were acquired with the same TR. Thus, T1 relaxation effects were uniform across all TEs.

The effects of transverse (T2) relaxation on the TE‐modulated edited 2HG peak were investigated according to the equation:

(1) $$S_{\mathrm{iv}}\left(\mathrm{TE}\right)=S_{s}\left(\mathrm{TE}\right)e^{\frac{-\mathrm{TE}}{T_{2,\mathrm{iv}}}}$$

Where $S_{\mathrm{iv}}\left(\mathrm{TE}\right)$ is the predicted in vivo 2HG signal as a function of TE, $S_{s}\left(\mathrm{TE}\right)$ is the simulated 2HG signal as a function of TE, and T_2,iv_ is the estimated in vivo T2 relaxation constant. $S_{\mathrm{iv}}\left(\mathrm{TE}\right)$ was evaluated with *T*_2,iv_ = 160 ms and *T*_2,iv_ = 200 ms, the upper and lower bounds of in vivo T2 for Glx.^14^, ^15^, ^16^, ^17^ $S_{\mathrm{iv}}\left(\mathrm{TE}\right)$ was also evaluated with *T*_2,iv_ = 264 ms, the T2 for 2HG which was estimated herein from in vivo 2HG‐edited acquisitions at multiple TEs and fit across all patients at once.

### 2.2 In Vivo

Fourteen patients with suspected gliomas (female, *n* = 7; male, *n* = 7; mean age, 38 ± 9.6 years) gave informed written consent with local Institutional Review Board approval. Eleven had surgical biopsies that confirmed IDH mutations, and three had lesions radiographically‐consistent with a low‐grade glioma. Data were acquired on a Philips Achieva 3T with a 32‐channel receive head coil. FLAIR images were acquired to identify tumor regions for voxel placement. MRS data were collected using a MEGA‐PRESS sequence with prospective frequency correction every 22 transients.^24^ Other parameters included second order projection‐based (PB)‐auto shimming, VAPOR water suppression^25^ with automatic water suppression optimization performed for each TE for the same patient, TR = 2 s, an edit‐ON frequency = 1.9 ppm, an edit‐OFF frequency = 7.5 ppm, 15‐ms editing pulses, 352 transients, 2 kHz spectral width and 2048 points. The voxel varied in location and ranged from 12 to 43 mL in volume, depending on the tumor size. The transmit frequency was set to 4.0 ppm (near the targeted 4.02 ppm 2HG resonance). Data were acquired with a TE of 90 ms for all participants, a TE of 70 ms for all but one participant due to time constraints from needing to scan two lesions, and a TE of 120 ms for eight participants. Data were preprocessed using Gannet 3.1^26^ with HLSVD water filtering, retrospective phase and frequency correction in the time domain,^27^ and manual phase and global frequency shift correction when needed. The 2HG‐edited spectra were fit with ProFit‐1D^28^, ^29^ with a basis set containing 2HG, glutamate, glutamine, NAA, N‐acetylaspartate glutamate, gamma‐aminobutyric acid (GABA) and glutathione.

To gauge the quality of the spectral fits, the fit‐quality number (FQN) of the 3.45–4.25 ppm range, which contains the 4.02 ppm 2HG peak and adjacent 3.75 ppm Glx peak (2HG‐Glx FQN), and the overall FQN of the entire fit range from 1.75 to 4.3 ppm were evaluated. The FQN was defined as the ratio between the variance of the fit residual divided by the variance of spectral noise.^28^ The linewidth and SNR were estimated to gauge spectral quality. SNR was calculated as the fitted NAA peak in the difference spectrum divided by the SD of the noise between 10.5 and 12.5 ppm. The integral of the absolute residual water signal from 4.25 to 6.5 ppm and the lipid signal from 1.05 to 1.9 ppm were evaluated to gauge the intensity of these nuisance signals. 2HG concentrations were also estimated relative to water as institutional units (i.u.) using Gannet 3.1 but with an estimated in vivo 2HG T2 relaxation time = 264 ms and water T2 relaxation time = 110 ms as measured here.

### 2.3 T2 Estimation

The T2 relaxation constant of 2HG was estimated using a previously described method.^30^ Briefly, the intrinsic TE‐dependence of the edited 2HG signal (generated from simulations) weighted by a transverse relaxation term was fit to the in vivo 2HG signal acquired at three TEs using a linear least squares fit in MATLAB and an estimated T2 relaxation time of Glx (180 ms) in healthy adults^14^, ^15^, ^16^, ^17^ as a starting value. The same process was performed to estimate the in vivo T2 relaxation time of Glx. The in vivo T2 relaxation time of water was estimated by fitting a monoexponential function $M_{\mathrm{xy}}\left(\mathrm{TE}\right)=M_{0}e^{\frac{-\mathrm{TE}}{T_{2,\mathrm{iv}}}}$ (Eq. 2) to the water area at each TE.

### 2.4 Spectral Overlap

To evaluate the effect of spectral linewidth on 2HG fitting accuracy at TEs 70 and 90 ms, 2HG‐edited spectra were simulated with concentrations extracted from LCModel fits to TE 70 ms edit‐OFF spectra with the exception of 2HG, which was set to 1 mM. The 2HG‐edited data were simulated with a default 7‐Hz or 13‐Hz line broadening and a base SNR = 150. Spectra were also simulated with possible perturbations, including 15 zero‐order phase from −35° to 35°, 15 SNR from 35 to 160, and 12 spectral baselines extracted from cubic spline fits to previously‐acquired GABA‐edited spectra.^31^, ^32^

### 2.5 Statistical analysis

Statistical analyses were performed in MATLAB. Two‐tailed paired Student's t‐tests were performed to assess whether differences in FQNs, fitted 2HG integrals and amplitudes, residual water integrals, and lipid integrals between TEs were statistically significant (*p*‐value < 0.05).

## 3 RESULTS

Figure 1B shows the spatial simulations for 4.02 ppm 2HG resonance in the edit‐ON (left), edit‐OFF (middle), and difference (DIFF, right) spectra with a TE of 90 ms. In the horizontal direction (first refocusing pulse), no spatial dependency of the 2HG multiplet was seen due to the short TE1 duration. In the vertical direction (second refocusing pulse), two regions can be seen in the OFF spectra where the multiplets are negative as desired in the green region and positive in the red region. Conversely, the ON multiplets are positive across the voxel. Thus, subtracting the two results in positive signal in the green region but substantially reduced signal in the red region, as previously described for GABA and GSH.^11^, ^33^ These red regions are where the 4.02 ppm spins underwent the second slice‐selective but the ˜1.9 ppm spins did not due to chemical shift displacement effects. With a slice‐selective bandwidth of 1264 Hz, this results in a chemical shift displacement effect of 21% and a certain degree of signal loss for the 4.02 ppm resonance over the entire voxel. Figure 1C shows the TE‐evolution of the 4.02 ppm 2HG multiplet in the edit‐ON, edit‐OFF, and DIFF case. In the edit‐OFF, the 2HG‐edited peak modulates with TE due to J‐coupling evolution. In the edit‐ON‐case, coupling is refocused and a similar multiplet can be seen at all TEs; some modulation of the 2HG peak can be seen in the edit‐ON due to evolution of the H_3_‐H_3_' strong coupling effects. Subtracting the OFF from the ON results in an edited 2HG resonance which reaches a maximum when the TE = 90 ms. This was confirmed in Figure 1D where the edit‐ON integral subtly decreases with increased TE while the edit‐OFF integral reaches a minimum at TE = 100 ms. As a result, the edited 2HG peak reaches a maximum at TE 90–110 ms with an integral that is ˜20% higher than at 70 ms. Although the edited 2HG peak still reaches a maximum at TE 90 ms with an estimated in vivo T2 relaxation constant of 264 ms, the benefit of TE 90 ms over TE 70 ms is reduced to 10%. With estimated Glx T2 relaxation constants of 160 and 200 ms, the edited 2HG peak remains maximal at TE 90 ms.

**FIGURE 2:**
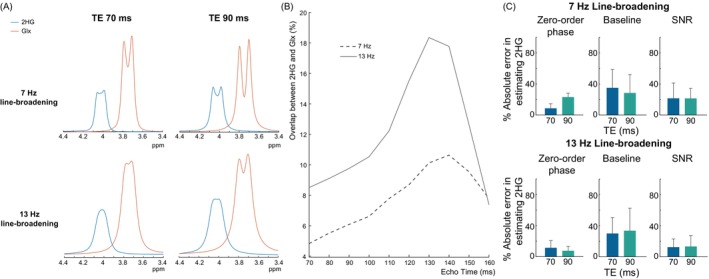
(A) Simulated edited 2HG and co‐edited Glx multiplets at 4.02 ppm and 3.75 ppm, respectively, in the difference spectra for TE 70 ms and 90 ms with different amounts of line‐broadening: 7‐Hz (top row) and 13‐Hz (bottom row) representing 2 different shimming conditions. At both TEs, the overlap between 2HG and Glx is greater as the line‐broadening gets larger. This overlap is slightly greater at TE 90 ms than at TE 70 ms. (B) Percent overlap between 2HG and Glx as a function of TE. At both 7‐Hz and 13‐Hz linebroadening, the percent overlap increases as the TE increases up to 130–140 ms. (C) Percent absolute error in estimating 2HG with changes in zero‐order phase, baseline, and SNR. Both TEs perform equally well in estimating 2HG with 7‐Hz and 13‐Hz linebroadening.

Figure 2 shows the overlap between 2HG and the co‐edited Glx as a function of TE. Figure 2A shows the simulated 4.02 ppm 2HG peak and co‐edited 3.75 ppm Glx peak at TE 70 and 90 ms with 7‐Hz linebroadening (top) and 13‐Hz linebroadening (bottom). With 7‐Hz linebroadening, 2HG is separated from Glx at both TEs, though the overlap at TE 90 ms is slightly more than that at TE 70 ms. With 13‐Hz linebroadening, this spectral overlap is increased for both TEs, though the relative spectral overlap is maintained between TEs. This is confirmed quantitatively in Figure 2B which shows the overlap between 2HG and Glx as a percentage of the total 4.02 ppm 2HG and 3.75 ppm Glx area. The percent spectral overlap increases with TE until 130–140 ms where it then decreases. At 7‐Hz line‐broadening, the spectral overlap with 7‐Hz is low at 4.8% and 6.1% for TE 70 ms and TE 90 ms, respectively. With 13‐Hz line broadening, the percent spectral overlap increases for all TEs especially at TEs 110–140 ms. This resulted in percent spectral overlaps of 8.5% for TE 70 ms and 9.8% for TE 90 ms. These differences in the spectral overlap between the two TEs have little effect on the 2HG fit accuracy across different spectral perturbations. With 7‐Hz linebroadening, the fit accuracy was slightly better at TE 70 ms with 3% higher fit accuracy at TE 70 ms than at TE 90 ms. With 13‐Hz linebroadening, the fit accuracy was equivalent between the two TEs with 0.2% higher fit accuracy at TE 70 ms than at TE 90 ms.

**FIGURE 3:**
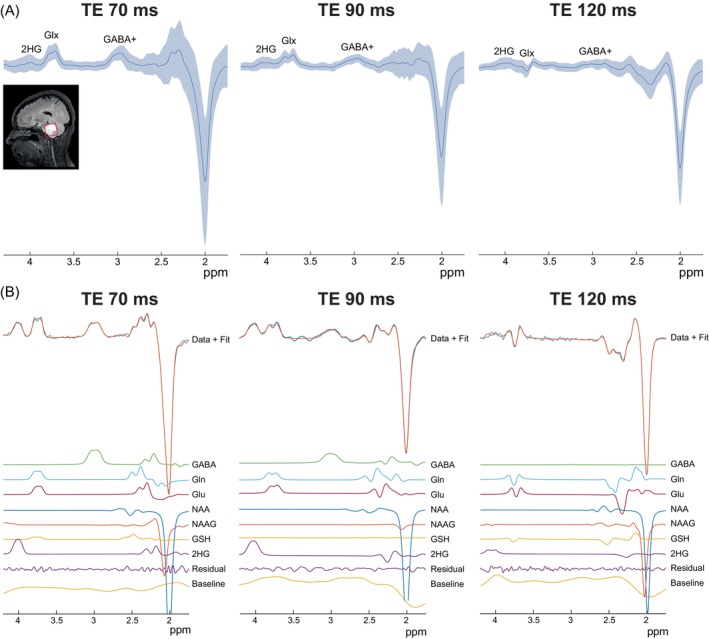
(A) Example tumor voxel placement with a size of 3 × 3 × 3 cm^3^ overlayed on a T2‐weighted FLAIR image as well as the average in vivo spectra (dark blue) plus and minus one SD (light blue) across patients at each TE (*N* = 14 for TE 90 ms, *N* = 13 for TE 70 ms, and *N* = 8 for TE 120 ms). (B) Representative fits to the 2HG‐edited spectra showing high‐quality fits for all three TEs.

In vivo 2HG‐edited spectra are shown in Figure 3. Figure 3A shows the average spectra (dark blue) plus and minus 1 SD (light blue) across patients at each TE. It can be seen that although high‐quality spectra can be observed at each TE, the spectra acquired at a TE of 70 ms show greater variability between patients at 1.75–2.5 ppm than at the other TEs. Figure 3B shows representative fits to the data. Overall, 2HG‐edited spectra and their corresponding fits were of high quality (Figure 3A). FQNs, linewidths, and SNR at each TE are reported in Table 1.

**TABLE 1:** FQNs as well as spectral linewidths and SNRs calculated from the NAA and 2HG peaks at all three TEs.

Parameter	TE 70 ms (*n* = 13)	TE 90 ms (*n* = 14)	TE 120 ms (*n* = 8)
NAA linewidths (Hz)	6.8 (3.4)	7.3 (4.4)	6.1 (1.7)
NAA SNRs	184 (227)	184 (205)	231 (113)
FQNs	16 (13.8)	9.7 (9.9)	13.1 (13.4)
2HG linewidths (Hz)	17.1 (5.6)	21 (3.7)	25.4 (1.2)
2HG SNRs	5 (17.1)	7.24 (8.2)	7.3 (15.6)
2HG FQNs	6.1 (15.8)	3.4 (5.8)	14.6 (23.3)

*Note*: Values are expressed as median (interquartile range).

**FIGURE 4:**
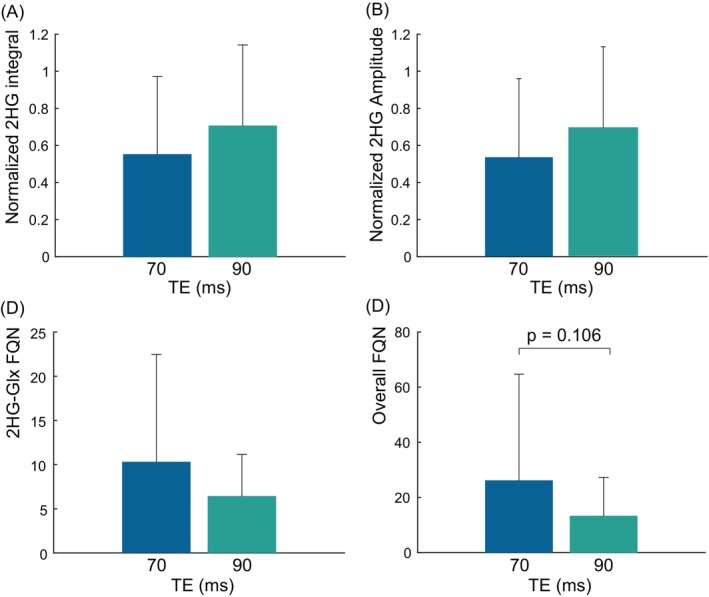
Bar plots of the (A) normalized 2HG integrals, (B) normalized 2HG amplitudes (C) 2HG‐Glx FQNs, and (D) overall FQNs are shown across all patients with data acquired at both TEs of 70 ms and 90 ms (*N* = 13). 2HG integrals are highest at a TE of 90 and 2HG‐Glx FQNs and overall FQNs are lowest at a TE of 90 ms.

2HG integrals and amplitudes normalized by the maximum value across TEs for each participant and 2HG concentrations are shown in Figure 4A,B. 2HG‐Glx FQNs, and overall FQNs in data acquired at TE 70 ms and TE 90 ms are shown in Figure 4C,D. Altogether, 2HG integrals at TE 90 ms were 23% higher than that at TE 70 ms, while the average fitted 2HG amplitudes at TE 90 ms were 30% higher than that at TE 70 ms. These differences were not statistically significant. 2HG concentrations were estimated to have an average of 2 i.u. for TE 70 ms and 2.62 i.u. for TE 90 ms. 2HG‐Glx FQNs were 38% lower at TE 90 ms than at TE 70 ms, and overall FQNs were 49% lower at TE 90 ms than at TE 70 ms. The differences in 2HG‐Glx FQNs were not statistically significant. There was, however, a trend towards significance for differences in overall FQN with a *p* = 0.1064. The overall FQNs were also lower at a TE of 90 ms than at a TE of 70 ms in the vast majority (77%) of patients. Fitted GABA and Glx amplitudes normalized by the maximum are shown in Supporting Information Figure S1. Fitted GABA amplitudes were 17% lower at a TE 90 ms than at a TE 70 ms (Figure S1A), but this was not statistically significant. There were no statistically significant differences in fitted Glx amplitudes (Figure S1B).

**FIGURE 5:**
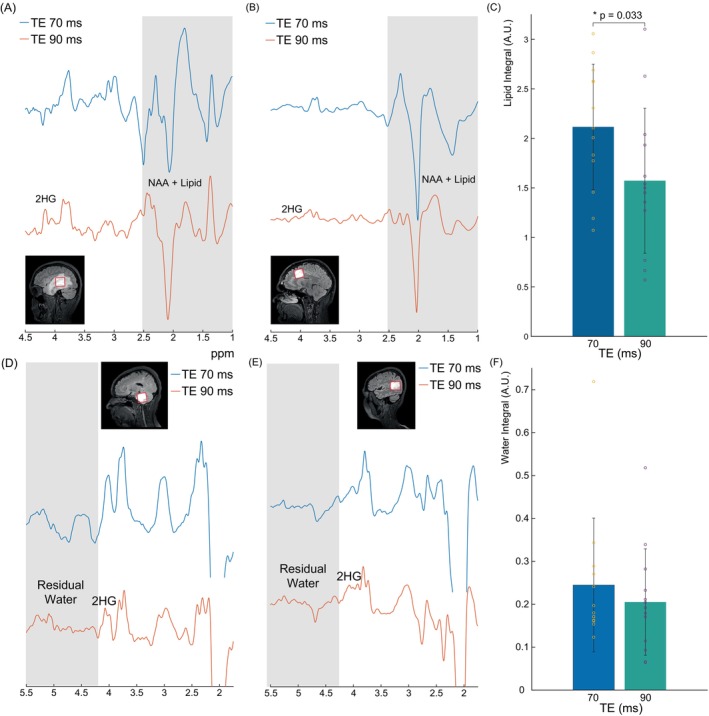
In vivo spectra at a TE of 70 ms and 90 ms from a voxel with a size of 3 × 3 × 3 cm^3^ placed in the center of a glioma located in the right hemisphere of one patient showing (A) a worst‐case scenario where substantial residual lipid can be seen in the 1–2.5 ppm range and (B) an average‐case scenario where less residual lipid can be seen. In both cases, however, it can be seen that there is lower lipid contamination at a TE of 90 ms than at a TE of 70 ms. (C) Bar plots of the lipid integrals showing a 26% lower lipid integrals at a TE of 90 ms than at a TE of 70 ms (*N* = 13). Spectra at a TE of 70 ms and a TE of 90 ms from a glioma voxel located in the brainstem of one patient showing (D) a worst‐case scenario where moderate residual water can be seen in the 4.1–5.5 ppm range and (E) an average‐case scenario where very little residual water can be seen. In the worst‐case scenario, however, it can also be seen that the residual water is lower at a TE of 90 ms than at a TE of 70 ms. (F) Bar plots of the water integrals showing 16% lower water signal at a TE of 90 ms than at a TE of 70 ms (*N* = 13). Lipid and residual water intensities are expressed in arbitrary units (A.U.) and do not include values from a patient without data acquired at TE 70 ms (due to time constraints). This was done for a fair comparison. Individual data points are plotted as yellow circles for TE 70 ms and as purple dots for TE 90 ms. The spectra within each subfigure (5A, 5B, 5D, and 5E) are from the same patients, while each subfigure features spectra from different patients. Altogether, four patients' spectra are featured in 5a, 5b, 5d, and 5e.

Lipid and water integral values at TE 70 ms and TE 90 ms and spectra showing example worst‐case and average‐case scenarios of lipid contamination and residual water. It can be seen in Figure 5A,B that the lipid signal (gray) has a significantly higher intensity at TE 70 ms than at TE 90 ms and overlaps with part of the metabolite spectra including the total NAA peak at 2.0 ppm. This is shown quantitatively in Figure 5C where it can be seen that the lipid integral is 26% higher at TE 70 ms than at TE 90 ms. This difference was statistically significant with a *p* = 0.033. In Figure 5D,E, it can be seen that the residual water signal (gray) adjacent to the edited 4.02 ppm 2HG signal is somewhat higher at TE 70 ms than at TE 90 ms in the worst‐case scenario. This is shown quantitatively in Figure 5F where it can be seen that the residual water integrals are 16% higher at TE 70 ms than at TE 90 ms. This difference was not statistically significant.

**FIGURE 6:**
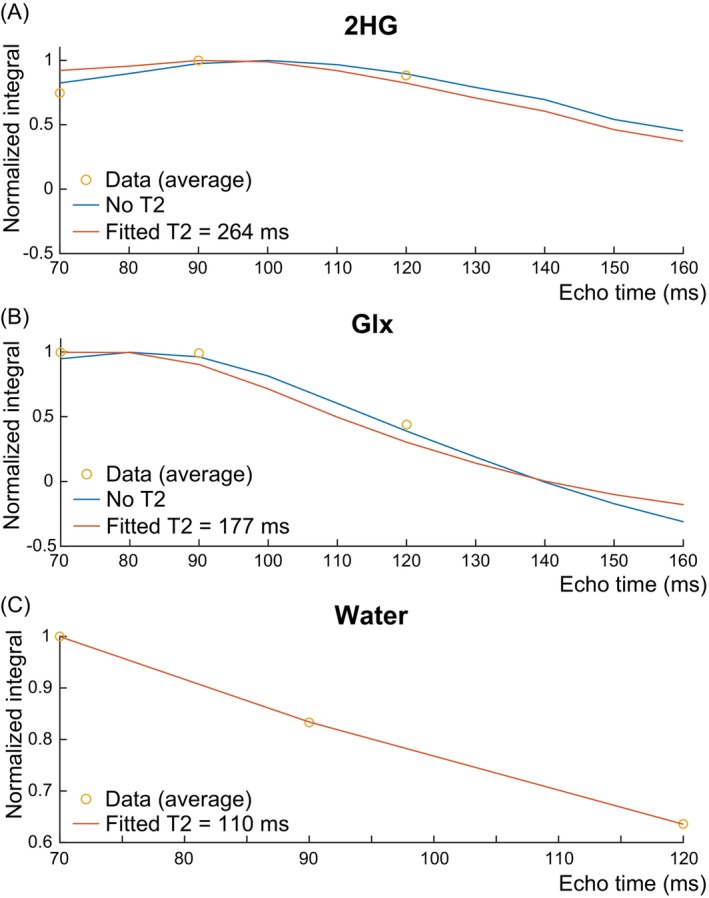
Integrals of the simulated (A) 2HG and (B) Glx peaks are shown with (orange) and without (blue) the fitted relaxation constants of 264 ms for 2HG and 177 ms for Glx (*N* = 8). The fitted water integrals with a T2 relaxation constant of 110 ms are also shown in (C) for TEs of 70, 90, and 120 ms (*N* = 8). Mean in vivo integrals are also shown as yellow circles. All integrals are normalized relative to the maximum integral value across TEs.

Figure 6 shows the fitted T2 curves to 2HG, Glx, and the tissue water signal from different tumor components and the estimated T2 values. The T2 of 2HG was estimated to be 264 ms while the T2 of Glx was estimated to be 177 ms, consistent with previously reported T2 values of 160–200 ms for Glu/Gln in healthy participants at 3T.^14^, ^15^, ^16^, ^17^ The water T2 relaxation constant was estimated to be 110 ms, longer than previously reported water T2 values in the brain of healthy participants, which ranged from 39 to 88 ms,^34^, ^35^ and consistent with previously reported water T2 values in brain tumors, which ranged from 86 to 181 ms.^36^ The error in the fitted T2 model relative to the average 2HG integrals across all three TEs had a mean ± SD of 5.6% ± 15.8% for 2HG with an *R* ^2^ of 0.7 and 13.2% ± 15.9% for Glx with an *R* ^2^ of 0.998.

**FIGURE 7:**
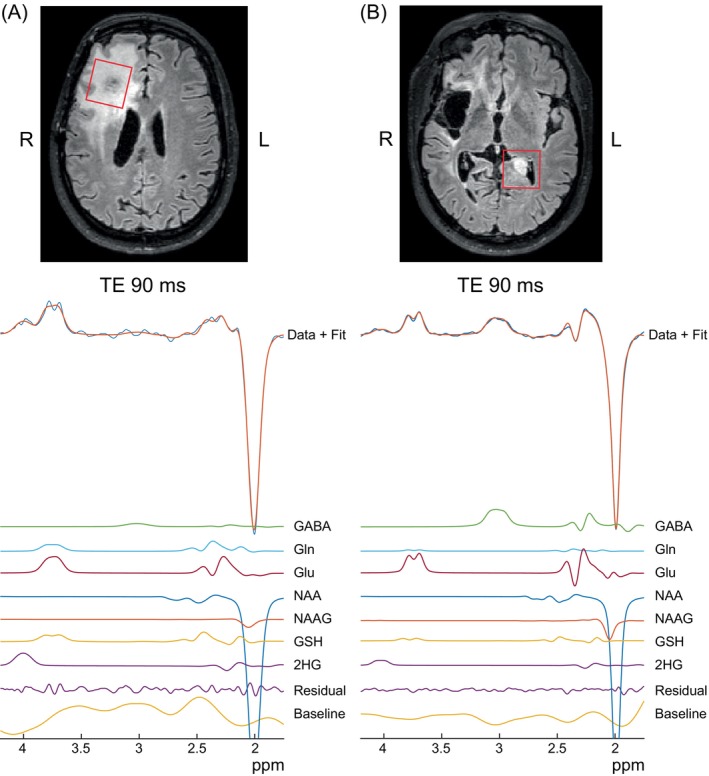
Representative in vivo spectra at a TE of 90 ms from a (A) voxel placed in the original lesion in the right hemisphere and (B) newly‐identified lesion in the contralateral (left) hemisphere. 2HG was fit in both lesions with concentrations greater than 1 i.u. thus confirming the progression of the glioma.

2HG‐edited spectra acquired at a TE of 90 ms and corresponding fits from two T2‐hyperintense regions in one patient are shown in Figure 7. The NAA SNR and linewidth of the spectrum in the originally identified lesion in the right hemisphere (Figure 7A) were 161 and 7.3 Hz, respectively, while the NAA SNR and linewidth of the spectrum in the new mass on the left hemisphere were 207 and 6.3 Hz, respectively (Figure 7B). The NAA SNR was higher in the left hemisphere lesion than that from the right hemisphere, likely due to the lower tumor content (and hence, higher NAA) in the left hemisphere lesion. The 2HG SNR and linewidth of the spectrum in the right hemisphere lesion (Figure 7A) were 10.8 and 22 Hz, respectively, while the 2HG SNR and linewidth of the spectrum in left hemisphere lesion (Figure 7B) were 5.7 and 20.5 Hz, respectively. The FQNs for the spectral fits were low at 1.86 and 2.75 for the original and new mass, respectively, indicating high‐quality fits. 2HG was measured in both lesions with concentrations of 1.32 and 1.18 i.u. in the original and new mass, respectively. The 2HG concentration in the new mass is larger than 60% of the 2HG concentrations measured from patients indicating progression of the glioma.

## 4 DISCUSSION

Here, the in vivo T2 relaxation time of 2HG, the TE dependence of the edited 2HG signal, and its separation from co‐edited Glx and nuisance signals using a MEGA‐PRESS sequence were investigated. The in vivo T2 relaxation time of 2HG was estimated to be long at 264 ms. Thus, a TE of 90 ms was found to produce the best results with maximization of the 2HG signal and minimization of residual water and lipid resonances. Although a TE of 90 ms did not change the co‐edited Glx signal, it did result in a slightly lower GABA signal.

In vivo detection of 2HG is challenging due to its coupled spin system, overlapped spectrum, heterogeneous tumor tissue which leads to difficult shim conditions, and low concentration especially in gliomas with low tumor cellularity.^5^ Thus, optimization of acquisition parameters for 2HG‐editing is important as it can allow for improved sensitivity and quality and thus, improved reproducibility. Clinically, this is also important for non‐invasively diagnosing patients for whom performing a biopsy presents a significant risk of neurological injury and catching the early progression of the glioma and response to treatment without having to re‐biopsy the lesion. In addition to providing high‐quality 2HG‐edited spectra, a TE 90 ms allowed for the detection of 2HG in both lesions of one patient thus confirming the progression of the tumor into the contralateral hemisphere.

Here, editing was performed with refocusing pulses that had a moderate bandwidth of 1.3 kHz and resulted in a ˜21% signal loss due to chemical shift displacement effects between the edited (1.9 ppm) and detected (4.02 ppm) resonances of 2HG.^37^ This loss could be reduced with the use of broadband pulses^38^ in an inner volume saturated MEGA‐PRESS sequence^39^ or MEGA‐sLASER, which contains adiabatic refocusing pulses with larger bandwidths and thus higher SNR due to reduced chemical shift displacement effects. The MEGA‐sLASER sequence, however, is prone to spurious echo artifacts due to the high number of unwanted coherence pathways.^40^, ^41^, ^42^ 2HG detection would be especially impacted by these artifacts as unsuppressed water is the main contributor to these spurious^43^ echoes, and the detected 4.02 ppm peak is in close proximity to water at 4.68 ppm.^40^ Also, the significant tissue heterogeneity present in tumors can result in poorer shim quality, which increases the likelihood of spurious echoes. Thus, a highly‐optimized crusher gradient and phase cycling scheme^43^ are needed to minimize unwanted coherence pathways when using sLASER localization to detect 2HG.

Despite the distance of many of these voxels from the scalp, it was found that the 2HG‐edited spectra contained a moderate amount of lipid; this is likely due to the abnormal lipid metabolism in gliomas compared to normal tissue which results in increased lipid content.^18^ Thus, the lipid resonances at ˜1.3–2.5 ppm^44^, ^45^ are saturated when the edit‐ON pulse is played at 1.9 ppm but not when the edit‐OFF pulse is played at 7.5 ppm, leading to a lipid signal in the 2HG‐edited spectrum. This is consistent with prior work which have reported elevated lipids in brain tumors with significant peaks at ˜2.25 ppm.^46^

Although this lipid signal does not overlap with the edited 4.02 ppm 2HG peak, it does overlap with the 2.0 ppm NAA peak which is often needed by spectral fitting software to determine starting values for the zero‐order and first‐order phases and global frequency shifts fitting. These lipid signals also overlap with the edited 2.22 ppm 2HG resonance, thus adding a confound for spectral fitting. Although this 2.22 ppm resonance is not the main 2HG peak targeted by the 2HG‐editing sequence, this resonance is partially inverted by the edit‐ON pulse at 1.9 ppm, but not the edit‐OFF, leading to a detectable peak at 2.22 ppm that contributes to the edited 2HG fit. Additionally, despite only being partially inverted by the edit‐ON pulse, the 2.22 ppm resonance has nearly the same magnitude as the 4.02 ppm resonance since the 2.22 ppm —CH_2_ group contains two protons, one more than that of the —CH group corresponding to the 4.02 ppm resonance. Thus, the increase in lipid combined with a substantial decrease in NAA in tumors can complicate spectral fitting of the edited 2HG signal similar to what was reported previously when using TE‐optimized PRESS in tumors.^46^ It was found, however, that the lipid signals are moderately reduced (˜26%) at the longer TE of 90 ms than at the shorter TE of 70 ms. This is likely due to T2 relaxation, which is especially short at ˜60 ms for lipids and results in a rapidly decaying signal that decreases significantly with increasing TE.^47^ Assuming a T2 relaxation time of 59 ms for lipid at 3T, the theoretical reduction in lipid at TE 90 ms vs. TE 70 ms (Eq. 2), is 29%, in agreement with the 26% decrease measured here. It should also be noted that, when performing 2HG‐editing, the lactate signal is subtracted out. Thus, the measured signal from 1.05 to 1.9 ppm can be attributed to lipid with high certainty.

It was observed here that the residual water was somewhat larger at TE 70 ms than at TE 90 ms. This is possibly due to greater T2 relaxation of water at the longer TE of 90 ms than at the shorter TE of 70 ms but to a lesser degree than lipid as the T2 relaxation time (110 ms) is longer than that of lipid. With this T2 relaxation time, the residual water is expected to be 16.6% smaller at TE 90 ms than at 70 ms, agreement with the 16.2% reduction found here. Although this decrease is moderate relative to lipid, the 4.68 ppm water peak is in close proximity to the edited 4.02 ppm 2HG resonance and can thus adversely affect the 2HG fit, especially in conditions where B_0_ shimming is difficult, such as when the tumor is highly heterogeneous, the tumor is near an air‐tissue interface, and/or the tumor is adjacent to metal implants from prior surgical resections. It should be noted that an automatic water suppression optimization was performed for each participant. In the water suppression optimization, the numerically optimized ratios of the flip angles for all VAPOR water suppression pulses were kept the same between patients, and only the global scaling was changed. Thus, the automatic water suppression optimization mainly considers local transmit field strength differences. Regardless, the difficult B0 shimming conditions in these patients likely shift part of the water resonance out of the bandwidth of the water suppression pulses, resulting in baseline distortions near the 4.02 ppm 2HG peak. These baseline distortions from water would be affected by T_2_ relaxation independent of the water suppression optimization performed during the scan.

2HG simulations over a range of TEs of 70–160 ms at increments of 10‐ms also revealed that the 4.02 ppm resonance reaches a maximum at a moderately long TE of 90–110 ms when T2 relaxation is not considered. When an in vivo T2 relaxation time of 264 ms, as measured here for 2HG, is considered, the TE‐modulated 2HG integral curve shifts towards shorter TEs. However, because of the long T2 relaxation time, this shift is not large enough to change the TE at which the 4.02 ppm resonance reaches a maximum, and a TE of 90 ms remains optimal for detecting 4.02 ppm resonance at its maximal value. Consistent with these simulation results, a TE 90 ms was also found to result in a higher (23%) 2HG signal integral than at TE 70 ms in vivo. This difference in 2HG signal integral is larger than the theoretical 10% predicted from the spatial simulations, and may be due to the significantly increased residual water and lipid present at the shorter TE of 70 ms, which could have led to the poorer spectral fits, as demonstrated by the higher FQNs, and consequently, underestimation of 2HG. Taken together, this higher 2HG signal integral, significant reduction in lipid, and moderate reduction in residual water at TE 90 ms than at TE 70 ms may be responsible for the better spectral fits at TE 90 ms than at TE 70 ms, as represented by the lower FQNs at TE 90 ms than at TE 70 ms. Additionally, while simulations indicated a slightly greater overlap between 2HG and Glx at TE 90 ms relative to TE 70 ms, this overlap did not result in an increase in fit error. Thus, the longer TE of 90 ms does not result in an increase in difficulty in separating 2HG from Glx relative to a TE of 70 ms.

Here, the T2 relaxation times of 2HG, Glx, and water were estimated by fitting the TE‐modulated 2HG integral curve to the in vivo 2HG integrals acquired at three TEs, and it was found that Glx has a T2 relaxation time of 177 ms, which is consistent with what has been reported for healthy adults.^14^, ^15^, ^16^, ^17^ The T2 relaxation time of water was also found to be 110 ms, which is longer than the T2 relaxation times of 39–88 ms reported in healthy adults,^34^, ^48^ but is consistent with previously reported water T2 values in brain tumors, which ranged from 86 to 181 ms. The longer water T2 relaxation in brain tumors is also consistent with the high signal (hyperintensity) relative to healthy tissue seen on T2‐weighted images and reflects the increased water content.^36^ It should be noted that this study uses a double spin‐echo sequence (MEGA‐PRESS) to estimate water T2 relaxation values, while the cited studies used a multiple spin‐echo sequence. This choice of sequence may partially explain the observed difference in the water T2 relaxation with respect to the literature. 2HG was also found to have a long in vivo T2 relaxation time of ˜264 ms. Considering this T2 relaxation time in the simulated TE‐modulated 2HG integral curves results in a TE of 90 ms remaining optimal for detecting 2HG with a maximal signal integral.

It should be noted that the number of patients (*n* = 8) for this portion of the study is low for generating normative relaxation values. The primary aim of this study, however, was the TE optimization of 2HG detection using J‐difference editing, and the T2 of 2HG was estimated for the purpose of estimating the bias that the apparent in vivo T2 relaxation imparts on the TE‐modulated 2HG integral curve. Although the T2 relaxation time was estimated across all gliomas, various factors related to the microenvironment have been known to significantly affect water and metabolite T2 relaxation times including tumor grade,^49^ tumor type,^36^ and edema.^50^ Gliomas have also been shown to possess varying levels of cellularity,^5^ which likely also affect T2 relaxation times. Considering the large number of variables that can affect T2 relaxation times, future work will focus on systematically investigating the effect of these factors on apparent T2 relaxation times.

Here, signal maximization of the 2HG signal was focused on the 4.02 ppm resonance as it is the least overlapped of the two edited 2HG resonances (4.02 and 2.22 ppm) and likely contributes the most to the overall fit quality. However, the 2.22 ppm resonance likely still contributes to the fit and may partially explain the differences in signal increases at TE 90 ms versus TE 70 ms between the simulations and in vivo experiments.

It should be noted that no statistically significant differences in fitted 2HG amplitudes between a TE of 70 ms and a TE of 90 ms were found. This is likely due to the large physiological variance in these tumors relative to the relatively small theoretical gain in SNR estimated from the simulations (10%). The range of 2HG concentrations was found to range substantially, and over half of the tumors contained low 2HG concentrations of less than 1 i.u. Altogether, this likely masked the statistical significance.

## 5 CONCLUSIONS

A comprehensive study on detecting 2HG with J‐difference editing at a range of TEs was performed. Simulations and in vivo experiments suggest an optimal TE of 90 ms when taking into consideration in vivo T2 relaxation, maximal 2HG signal integral, and spectral overlap with co‐edited Glx, lipid, and residual water. The in vivo T2 relaxation times of 2HG, Glx, and water in gliomas were also estimated to be 264, 177, and 110 ms, respectively.

## FUNDING INFORMATION

This project was sponsored by the Cancer Prevention and Research Institute of Texas (CPRIT)/Grant number: RR180056.

## Supporting information


**TABLE S1.** Minimum Reporting Standards in Magnetic Resonance Spectroscopy checklist.
**FIGURE S1.** Bar plots of the normalized GABA amplitudes (a) and Glx amplitudes (b) across all patients with data acquired at both TEs of 70 ms and 90 ms (*N* = 13).
