# Supplementary material for: TE optimization for J‐difference editing of 2‐hydroxyglutarate at 3T
Source: Magn Reson Med. 2025 May 20;94(4):1363–76. doi: 10.1002/mrm.30561 (PMC12309877; doi:10.1002/mrm.30561)
Supplement: Supplementary file 1 — TABLE S1. Minimum Reporting Standards in Magnetic Resonance Spectroscopy checklist. FIGURE S1. Bar plots of the normalized GABA amplitudes (a) and Glx amplitudes (b) across all patients with data acquired at both TEs of 70 ms and 90 ms (N = 13). [file MRM-94-1363-s001.docx]

**Supporting Information Table S1** Minimum Reporting Standards in Magnetic Resonance Spectroscopy checklist

Hardware

| Field Strength | 3T |
| --- | --- |
| Manufacturer | Philips |
| Model (Software) | Achieva, software release 5.7.1 |
| RF Coil | 32-channel ^1^H head coil |
| Additional Hardware | N/A |

Acquisition

| Pulse Sequence | MEGA-PRESS |
| --- | --- |
| Volume of Interest (VOI) | Tumor (hyperintensity of a FLAIR image) |
| Nominal VOI size | 12 mL to 43 mL in volume depending on the tumor size |
| RF Coil | 32-channel head coil |
| Repetition Time (TR) and Echo Time (TE) | TR = 2 s and TE = 70, 90, and 120 ms |
| Number of averaged spectra | 352 averages |
| Additional Parameters | 2000 Hz spectral width, 2048 spectral points |
| Water Suppression Method | VAPOR |
| Shimming Method | Pencil beam (PB)-auto, 2^nd^ order |
| Triggering or Motion Correction | N/A |

Analysis and Data Quality

| Analysis Software | Gannet 3.1 |
| --- | --- |
| Processing steps deviating from reference | Manual phase and global frequency shift correction when needed |
| Output Measure | Institutional Units |
| Quantification Reference | Unsuppressed water |
| Reported Variables | SNR (based off the fitted NAA peak), full-width-at-half-max of the fitted NAA peak, integrals of the absolute value of the lipid and water resonances. |
| Data exclusion criteria | Visual inspection (lack of interpretable spectrum) |
| Sample spectrum | Figure 3a, Figure 5a, Figure 5b, Figure 5d, Figure 5e |

**Supporting Information Figure S1** Bar plots of the normalized GABA amplitudes (a) and Glx amplitudes (b) across all patients with data acquired at both TEs of 70 ms and 90 ms (N = 13).
